# Prevalence of celiac disease in multiple sclerosis

**DOI:** 10.1186/1471-2377-11-31

**Published:** 2011-03-07

**Authors:** Luis Rodrigo, Carlos Hernández-Lahoz, Dolores Fuentes, Noemí Alvarez, Antonio López-Vázquez, Segundo González

**Affiliations:** 1 Gastroenterology, Hospital Universitario Central de Asturias (HUCA), c/Celestino Villamil, s. n°., 33006. Oviedo, Spain; 2 Neurology, Hospital Universitario Central de Asturias (HUCA), c/Celestino Villamil, s. n°., 33006. Oviedo, Spain; 3 Immunology Services Hospital Universitario Central de Asturias (HUCA), c/Celestino Villamil, s. n°., 33006. Oviedo, Spain

## Abstract

**Background:**

Celiac disease (CD) is a common systemic disease related to a permanent intolerance to gluten and is often associated with different autoimmune and neurological diseases. Its mean prevalence in the general population is 1-2% worldwide. Our aim was to study the prevalence of celiac disease in a prospective series of Multiple Sclerosis (MS) patients and their first-degree relatives.

**Methods:**

We analyzed the prevalence of serological, histological and genetic CD markers in a series of 72 MS patients and in their 126 first-degree relatives, compared to 123 healthy controls.

**Results:**

Tissue IgA-anti-transglutaminase-2 antibodies were positive in 7 MS patients (10%), compared to 3 healthy controls (2.4%) (p < 0.05). OR: 5.33 (CI-95%: 1.074-26.425). No differences were found in HLA-DQ2 markers between MS patients (29%) and controls (26%) (NS).

We detected mild or moderate villous atrophy (Marsh III type) in duodenal biopsies, in 8 MS patients (11.1%). We also found a high proportion of CD among first-degree relatives: 23/126 (32%). Several associated diseases were detected, mainly dermatitis 41 (57%) and iron deficiency anemia in 28 (39%) MS patients. We also found in them, an increased frequency of circulating auto-antibodies such as anti-TPO in 19 (26%), ANA in 11 (15%) and AMA in 2 (3%).

**Conclusions:**

We have found an increased prevalence of CD in 8 of the 72 MS patients (11.1%) and also in their first-degree relatives (23/126 [32%]). Therefore, increased efforts aimed at the early detection and dietary treatment of CD, among antibody-positive MS patients, are advisable.

## Background

Multiple Sclerosis (MS) is a chronic disease of unknown etiology, characterized by the presence of disseminated demyelinating lesions in the central nervous system (CNS), and associated with autoimmunity. Activated, potentially autoimmune, T cells cross the blood-brain barrier and produce inflammatory plaques and axonal loss in the brain, spinal cord or optic nerves. The end result is the accumulation of gliosis and demyelination and areas in the CNS. MS affects about 1 ‰ of the population worldwide.

Mainly occurs in young people, more often women. The Relapsing-Remitting form of Multiple Sclerosis (RRMS) makes up 80% of the total number of MS cases and is characterized by intermittent episodes of relapses and prolonged remissions. Clinically, patients display episodes of acute neurological dysfunction, followed by recovery and a symptom-free interval until the next outbreak. These recurrent events eventually lead to more permanent neurological disabilities. Using an experimental model of autoimmune encephalitis as a starting point, immunomodulatory and immunosuppressive therapies have then proved effective in preventing relapses in MS patients, especially when performed early in the course of the disease [1-7].

Celiac disease (CD) is a systemic autoimmune disorder characterized by permanent intolerance to gluten in genetically predisposed individuals. The genetic basis for gluten intolerance is located in the region of chromosome 6 coding for HLA class-II [8-11]. Some patients with RRMS show high levels of anti-tissue transglutaminase-2 (TGt-2) antibodies, which is an important serological marker in the diagnosis of the disease [12]. Based on this observation and on the possible association of MS with other autoimmune processes, we have applied a specific protocol for the systematic assessment of CD in a population of RRMS patients.

## Methods

### Patients

We conducted a prospective observational study of a consecutive series of 80 patients suffering from well-established and clinically definite MS. They were previously diagnosed with RRMS and checked up at an outpatient clinic for demyelinating disorders within the Department of Neurology at the Central University Hospital of Asturias (HUCA). This is an urban tertiary hospital located in Northern Spain, serving an area with a population of 250,000. Patients were enrolled during a one-year period (January-December 2006). Of the initial 80 RRMS a total of 72, were included in this study (the other 8 didn't complete the study protocol).

MS patients with primary or secondary progressive forms of the disease (PP or SP) were not included in the study, because most of these patients were very physically disabled, in wheelchairs, and it would have been very inconvenient for them to attend the necessary check-ups.

We also included in this study a total of 126 first-degree relatives of the 72 RRMS patients. We compared the findings with a control group of 123 marrow blood donors of the same area.

The study was approved by the Research and Ethics Committee of the HUCA, following the principles included in the modified Declaration of Helsinki.

All RRMS patients in this series were diagnosed on the basis of medical history, neurological examination and paraclinical positive tests, including Magnetic Resonance Imaging (MRI), Cerebrospinal fluid (CSF) and Visually Evoked Potentials (VEP), according to the 2005 McDonald criteria [13]. All cases also met the spatial and temporal dissemination criteria.

### Clinical parameters

At the time of inclusion in the study, RRMS patients underwent a brain and spine MRI with intravenous injection of 0.2 ml/kg body weight of gadolinium (Magnevist™ 0.5 mmol/ml) as a contrast agent to assess uptake. All subjects were studied and monitored by the same neurologist (CHL), who followed-up with them at least twice a year.

With regard to treatment, 48 of the patients were receiving immunomodulatory therapy. Four patients received interferon beta-1a (30 mcg. IM/weekly), while 39 received interferon beta-1a (22-44 mcg. SQ/3 days per week) or interferon beta-1b (250 mcg SQ, on alternate days). Five other patients received glatiramer acetate (20 mg SQ/daily). No patient received immunosuppressive therapy. The remaining 24 patients were not treated.

During relapses, subjects received hospital inpatient treatment with methylprednisolone (500-1000 mg IV/day for 3-5 days), except those with sensitive outbreaks, who were treated as outpatients with oral prednisone at a dose of 1 mg/kg/day for 1 week. In both cases, the initial steroid therapy was progressively decreased throughout the treatment period.

### MS studies

MS disease activity was assessed by measuring the annual relapse rates and the EDSS (Expanded Disability Status Scale). The initial relapse rate value was calculated as the average over the two years preceding the admission into the study. EDSS was assessed at the time of admission into the study, leaving at least one month between the most recent episode and the EDSS assessment. The data on the number of relapses, their annual rate, and EDSS were updated at every follow-up visit, during the study period.

### CD studies

All patients were invited to participate on a voluntary basis in a screening test for CD associated with the diagnosed RRMS. In 90% of the cases, the patients accepted. These subjects were referred to an outpatient Gastroenterology clinic specialized in the study of small intestine diseases, which was located in the same hospital. All consenting patients were evaluated by the same gastroenterologist (LR). Every patient underwent a series of analytical assessments, which included serological and CD genetic markers, together with an upper GI endoscopy with multiple duodenal biopsies.

### Laboratory tests

Complete blood count tests including erythrosedimentation rates (ESRs) were performed with an automated hematology analyzer (Cell-DYN 3500 model R Abbott); complete coagulation panels were also carried out with a coagulation analyzer (ACL 3000, Menarini). Normal hemoglobin values were in the 12-14 g/dl range, and cases of iron deficiency anemia were defined according to WHO criteria, i.e. Hb < 13 g/dl in males and < 12 g/dl. in females. Normal white blood cell count was set between 4-10 × 10^3^/L and normal platelet count was between 130-400 × 10^3^/L.

Comprehensive analytical biochemistry tests were performed on the blood samples, including analyses of the following parameters: iron metabolism, including serum iron levels; transferrin saturation index (TSI) and serum ferritin. Normal values of serum iro were in the 60-140 mcg/ml range, whereas the normal ferritin values were 13-150 ng/ml. The TSI was considered normal, for values ranging between 25-45%.

We also assessed liver function tests (LFTs) including alkaline phosphatase (AP), aspartate aminotransferase (AST), alanine aminotransferase (ALT), gamma glutamyl transpeptidase (GGT) and bilirubin. The following measurements were also performed: total serum calcium, folate and vitamin B-12, serum creatinine, total cholesterol (with normal values in the 150-240 mg/dl range), high density lipoprotein (HDL), low density lipoprotein (LDL) and triglycerides, urea, glucose, total protein, albumin and acute phase reactants such as CRP (C-reactive protein). The normal values of AST and ALT were between 1-31 U/L. Immunoglobulins (IgG, IgA, IgM) were quantified by nephelometry, and circulating levels of the thyroid stimulating hormone (TSH, normal range: 0.25-5.0 mU/L) and the thyroid hormones (T3 and T4) were also evaluated. Finally, an urinalysis with microscopic examination of sediment was performed. Serum levels of triglycerides and total cholesterol were determined using enzymatic tests and values were expressed in mg/dl. All analytical studies were completed on a Hitachi Modular automated analyzer SXA-PPBD (Roche) using enzymatic or kinetic methods.

### Serological markers of autoimmunity

The presence of anti-nuclear antibodies (ANA) and anti-thyroid antibodies like anti-peroxidase (anti-TPO) were measured routinely, and in those cases with altered LFTs, the anti-mitochondrial antibody (AMA) was also assessed. The determination of ANAs, anti-TPO and AMAs, was conducted by indirect immunofluorescence assay on the Hep-20-10 cell line (Euro-Immun, Lübeck, Germany).

### Serological markers of celiac disease

Tissue transglutaminase 2 (TGt-2) anti-IgA antibody count was the only marker used; quantification was achieved with the aid of a commercially available ELISA kit (Phadia Diagnostics, Upsala, Sweden). We reported as positive those tests with values > 2 U/ml, a threshold considered to allow for a greater sensitivity in the general population [14], and which we had already confirmed in a previous comparative study of several commercial kits [15]

### Genetic markers

To assess genetic susceptibility to CD two HLA-class II markers, the HLA-DQ2 (DQA1*0501 and DQB1*0201) and the HLA-DQ8 (DQA1*0301 and DQB1*0302) were assayed, by using a Polymerase Chain Reaction (PCR) with a commercially available kit (Protrans^® ^HLA Celiac Disease Domino System, Protrans, Ketsch, Germany).

### Duodenal biopsy studies

Upon approval of informed consent procedures by the Hospital Ethics Committee, an upper endoscopy with multiple duodenal biopsies (at least 4) was performed in all the patients included in the study. Samples were routinely stained with Hematoxylin-Eosin (HE) and with immunohistochemical CD3-specific stains, which are routinely used to verify the presence of intraepithelial lymphocytes (IEL). IELs were in turn quantified per 100 epithelial cells.

Duodenal biopsies were studied by two expert pathologists and classified into the following types: Stage 0: Histologically normal duodenum; Stage 1: Increased IEL infiltration with a total count > 25% of epithelial cells; Stage 2: hyperplasia of crypts and/or diffuse chronic inflammatory infiltrate at the level of the lamina propria; Stage 3: villous atrophy, subdivided into three categories: a) mild, b) moderate, and c) severe, according to the anatomo-pathological classification for CD screening previously described by Marsh in 1992 [16] and later modified by Oberhuber *et al. *[17] in 1999.

We used neither gastric biopsies nor breath testing to routinely determine the presence of Helicobacter pylori infection in our patients.

### Statistics

Descriptive statistics were used on continuous parameters: calculation of means, standard deviations, and observed ranges. For qualitative variables, percentages were used in the analyses. We determined the Chi-square test for these categorical variables between non-paired groups, using the exact Fisher's test when necessary. The odds ratio and CI-95% were also calculated. A p value < 0.05, was considered as statistically significant.

## Results

Of the 72 RRMS patients included into the study, 60 (83%) were women, giving a female-male ratio of 5/1. Their ages ranged from 24 to 58 years old. The mean duration of RRMS, the clinical activity, the number of patients receiving any type of immunomodulatory treatment, and the percentage of fertility are shown in Table 1.

**Table 1:** Clinical and demographic characteristics of RRMS patients (n = 72)

Females, n (%)	60 (83)
Age in years, mean ± SD (range)	43 ± 10 (24-61)

RRMS duration in years, mean ± SD (range)	11 ± 6 (1-30)

Annual relapse rate, mean ± SD (range)	1.1 ± 0.4 (0-2)

EDSS*, mean ± SD, [median], (range)	1.7 ± 1.1, [2], (0-5)
Immunomodulatory therapy, n(%)	44 (67)

They have had children, n (%)	32 (53)

EDSS* = Expanded Disability Status Scale; SD = Standard Deviation

The clinical indicators of MS and the results of several paraclinical tests are presented, including the presence of oligoclonal bands (OCB) in the CSF, visual evoked potentials (VEP) and somato-sensorial evoked potentials (SSEP), together with MRI findings at the time of inclusion in the study (Table 2).

**Table 2:** Paraclinical tests of RRMS patients (n = 72)

CSF oligoclonal bands^1^, n (%)	51 (71)
Altered VEPs^2 ^+, n (%)	46 (64)

Altered SEPs^3^+, n (%)	52 (72.2)

T1-weighted gadolinium-enhanced MRI^4^ lesions, n^5 ^(%)	25 (35)

T2-MRI supratentorial lesions, mean ± SD (range)	4.6 ± 6.5 (6-30)

T2-MRI infratentorial lesions, mean ± SD (range)	1.6 ± 2.5 (0-10)

T2-MRI medullar lesions, mean ± SD (range)	1.2 ± 1.4 (0-6)

**^1^CSF = **Cerebrospinal fluid; **^2^VEPs = **Visual evoked potentials; **^3^SEP = **Somatosensory evoked potentials; **^4^MRI = **Magnetic Resonance Imaging; **n^5 ^**= number of cases

Overall, the mean values for the different blood chemistry analyses performed on the population were normal, showing a significant dispersion and wide ranges.

The frequency of serological and genetic markers was compared to the findings in the control group. Histological duodenal biopsies were made in only RRMS patients and their results are shown in Table 3.

**Table 3:** CD markers in RRMS and controls

CD^1 ^markers	RRMS (n = 72)	Controls (n = 123)	p
Increased anti-tTG2^2^, n [%]	7 (10)	3 (2.4)	< 0.05

HLA-DQ2^3 ^(+), n [%]	21 (29)	32 (26)	NS^5^

HLA-DQ8 (+), n (%)	8 (11)	17 (14)	NS

Villous atrophy (Marsh 3), n (%)	8 (11)	ND^4^	NS

**^1^CD = **Celiac Disease; **^2^Anti-tTG2 = **Anti-tissue transglutaminase-2 antibodies

^3^**HLA = **Human leukocyte antigens; **^4^ND = **Not done; **^5^NS = **Not significant

Age, sex, anti-TGt2 values, genetic and histologic characteristics of the eight cases diagnosed with simultaneous celiac disease, which showed slight increased serologic levels of TGt-2 and diverse degrees of villous atrophy are shown in Table 4.

**Table 4:** Demographic, serological, genetic and histological characteristics of the eight patients exhibiting an associated CD

Patients	Sex	Age	Anti-tTG2 (U/ml)	HLA-DQ2	HLA-DQ8	Marsh^2^
1	F^1^	33	187	(+)	ND^3^	3b

2	F	28	10.3	(+)	ND	3a

3	F	25	8.5	(+)	ND	3a

4	F	27	4.2	(+)	ND	3a

5	F	26	9.4	(+)	ND	3b

6	F	29	2.8	(+)	ND	3a

7	F	23	3.6	(-)	(+)	3a

8	F	34	1.8	(+)	ND	3a

**^1^F = **Female; **^2^Marsh = **Histological classification of duodenal biopsies for CD; **^3^ND **= Not done

The clinical characteristics of the eight patients with associated celiac disease refered to the presence of gastrointestinal symptoms were found in 6 (75%); weight loss was observed in 1 patient (12.5%). The main neurologic form was myelitis in 4 (50%) and mixed in 4 (50%). No ataxia forms of presentation were found. Three patients (37.5%) were treated with interferon-beta. We found 2 patients (25%) with ANAs (+). All these data are shown in Table 5.

**Table 5:** Clinical characteristics of the eight patients with associated CD

Patients	Gastrointestinal symptoms	Weight loss	Neurological syndrome	IFN-beta treatment	Dermatitis	ANA (+)
1	Diarrhea	Yes	Myelitis	No	Yes	No

2	None	No	Myelitis	Yes	No	Yes

3	GERD+Constipation	No	Optical neuritis	No	Yes	Yes

4	Constipation	No	Encephalitis	No	No	No

5	GERD	No	Myelitis	No	Yes	No

6	Constipation	No	Encephalitis	Yes	No	No

7	GERD+Constipation	No	Optical neuritis	No	No	No

8	None	No	Myelitis	Yes	Yes	No

**GERD **= Gastroesophageal Reflux Disease; **ANA = **Antinuclear antibody test

All the celiac patients were put on a gluten free diet and all of them improved considerably both with respect to the gastrointestinal and to the neurological symptomatology in the follow-up period.

The only differential parameter between celiacs and non-celiacs was the age of onset of MS, that was younger (35 ± 7 y.o) in the former and older (44 ± 10 y.o) in the latter (p < 0.05)

**Figure 1:**
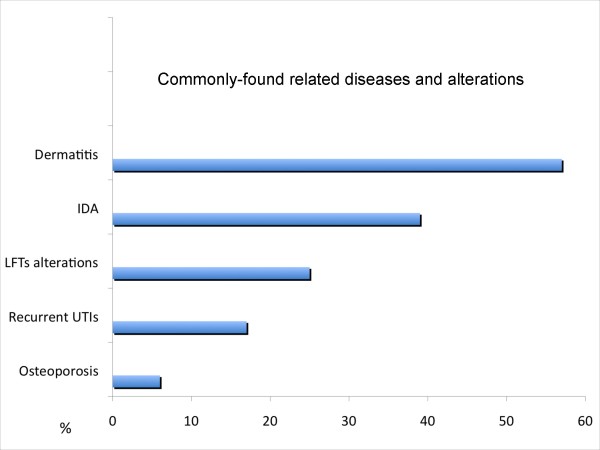
**Percentages of individuals who scored positive for several associated conditions are shown**. IDA = Iron Deficiency Anemia; LFTs = Liver Function Tests; UTIs = Urinary Tract Infections

We have observed a high frequency of several different related diseases. Dermatitis: 41 (57%); Iron deficiency anemia: 28 (39%); Liver function test alterations: 18 (25%); Recurrent urinary tract infections: 12 (17%); Osteoporosis: 4 (6%) (Figure 1).

It is significant that a high prevalence of CD was found in first-degree relatives of RRMS patients, with 23 (32%) positive cases among the 126 studied individuals.

**Figure 2:**
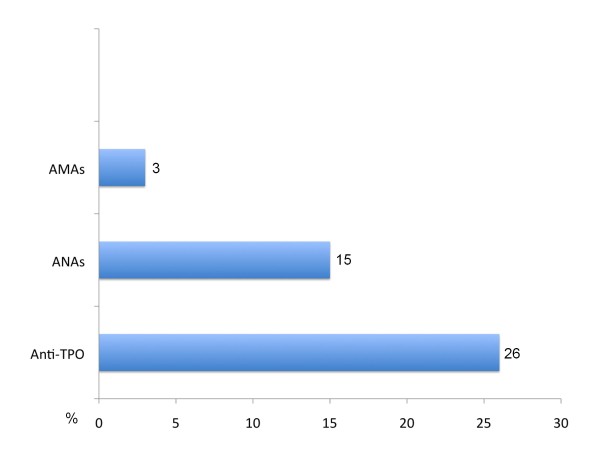
**Frequency of associated auto-antibodies found in all MS patients**. Anti-TPO = Anti-thyroid peroxidase antibodies; ANAs = Anti-nuclear antibodies; AMAs = Anti-mitochondrial antibodies

We also discovered a variety of associated circulating auto-antibodies in these patients, the most frequently observed being anti-thyroid peroxidase (anti-TPO) antibodies, followed by anti-nuclear antibodies (ANA), and, less frequently, anti-mitochondrial antibodies (AMA). Their percentages are shown in Figure 2.

## Discussion

Our data support the hypothesis that there is an increased prevalence of serological markers of CD in RRMS patients (OR: 5.33; CI-95%: 1.07-26.45). Nevertheless, we have not found any differences between the DQ2 and DQ8 genetic markers in the patient and control groups. We also discovered several duodenal lesions in 21 (29%) of RRMS patients and mild villous atrophy in 8 (11.1%) of them.

All of these findings, together with the high prevalence of CD in first-degree relatives (32%) support a frequent association with gluten intolerance in RRMS patients.

These results have been observed by other authors from Israel [18]. The specific role of serological gluten markers in the pathogenesis of multiple sclerosis remains uncertain. MS is a disorder presumed to be of autoimmune nature, characterized by a chronic demyelinating inflammatory process which affects the CNS, but its etiology remains unknown. MS affects mainly Caucasians, predominantly women, and in most cases, appears in young adulthood. Genetic, immunopathologic and environmental factors contribute to its complex multifactorial pathogenesis, via mechanisms that remain poorly understood [19].

Celiac disease (CD) is a systemic autoimmune disorder with a well-defined etiology. It appears as a consequence of permanent gluten intolerance, which occurs in genetically predisposed individuals. CD affects primarily, but not exclusively, the small intestine. It can start at any age, both during childhood or adolescence, and it often appears in adulthood [9]. Currently known genetic markers are excellent predictors of propensity for CD, but a small proportion of patients (5-10%) are negative for both DQ2 and DQ8, suggesting that other genetic markers of CD are yet to be described. Good candidates are additional HLA class I markers, including MICA and MICB, as well as other non-HLA markers detected by GWAS studies [20].

Until about two decades ago, CD was considered a rare disease, but it has now been shown to be very common, with a fairly uniform worldwide distribution and an average prevalence of 1-2%, although this disease is often underestimated, and therefore under-diagnosed [21].

Individuals predisposed to CD exhibit inappropriate immune response to peptides derived from wheat, barley, and rye prolamins. Although several immunogenic epitopes are present in gluten, the most powerful is a peptide of 33 amino acids (residues 57-89), contained in the α-gliadin fraction of gluten. This peptide is rich in proline and glutamine residues and appears to be primarily responsible for gluten toxicity, making it the main pathogenic factor linked to the occurrence of the disease [22].

During the pathogenesis leading to the appearance of lesions in the small intestine, gliadin peptides cross the intestinal epithelium and, at the level of the submucosa, they are modified by tissue transglutaminase (TGt2). Deamidated peptides are presented to dendritic cells with HLA-DQ2/DQ8 receptors, which in turn present them to CD4+ cells which, when activated, release cytokines such as Interferon-gamma (IFN-γ) and other mediators of inflammation. The characteristic histological changes, inflammation and/or villous atrophy, occur in the small intestinal mucosa [23,24].

In 1997, Dieterich *et al. *reported that tissue transglutaminase type 2 (TGt-2) is the self-antigen that reacts against endomysium antibodies (EMA). This must to be evaluated only by using indirect immunofluorescence technique on monkey esophageal epithelium. The determination of human antibody reactive against TGt-2 has the same diagnostic value as the EMA, but has a greater clinical utility, because it can be achieved with simpler techniques (commercial ELISA). Such diagnostic methods were found to allow for high sensitivity and specificity (close to 90%) in the various studies carried out in the early years after their commercialization [25].

As the determination of anti-TGt-2 antibodies became used more routinely, a marked decrease of sensitivity was found to exist when studying patients without villous atrophy (or with a mild degree, as normally happens in adults). Several published studies among large groups of celiac patients, have clearly demonstrated that a positive anti-TGt-2 antibody test, correlates linearly with the degree of severity of the histological duodenal lesions found. In patients presenting only lymphocytic enteritis (Marsh I), its sensitivity is very low, between 15-30% at best [26-28]. These findings have led to the application of new diagnostic strategies, such as the concurrent use of genetic testing, or the determination of anti-TGt-2 antibodies in duodenal aspirate in cases of negative serology. An alternative approach to increasing sensitivity relies on the use of a corrected threshold for positivity; its value has been recommended as 2 U/ml for adults [15,29]. This is the threshold that we have applied in the present study. We did not employ EMA antibodies, because they measure the same substract than the anti-TGt-2 and the anti-gliadin, because they have a lower sensitivity and specificity than the anti-TGt-2 used.

The findings from duodenal biopsies are still considered by most experts as the "cornerstone" or the "gold standard" for diagnosis of CD. However, this statement emphatic as it may sound, is currently being challenged, especially if we take into account the adult forms of CD. In cases with very high levels of anti-TGt-2 antibodies (>100 U/ml), the duodenal biopsy may be avoided, since in most cases (> 90%) such high levels are associated with the presence of villous atrophy [30]. When the histological results are normal, but the serology is positive and the clinical picture is suggestive of CD, the diagnostic uncertainty remains, and in such cases a gluten-free diet (GFD) could be recommended for at least 6 months, before definitely ruling out a CD.

When this diagnosis is dubious, it could be useful to perform a genetic and familial study such as those we carried out in the present study. While the prevalence of DQ2 in the general population is 20-40%, it reaches 90% among celiac patients, as confirmed in a study by Fernández-Bañares *et al.*, in which a series of patients with presumed irritable bowel syndrome and predominant diarrhea were screened for possible CD [31]. The determination of the known genetic markers for celiac disease (DQ2 and DQ8) therefore, has a high negative predictive value.

In the present study we have found a high prevalence of celiac disease among MS patients (11.1%) based mainly on the presence of villous atrophy in the duodenal biopsy. This is between 5-10 times higher that the frequency found in the general population. All the 8 celiac patients were female. Six had gastrointestinal symptoms, with predominant constipation in 5 and chronic gastro-esophageal reflux disease (GERD), in 3. Surprisingly only one patient had chronic diarrhoea and weight loss. This form is called "atypical presentation" and is common in adults, in contrast to the "classical form" that predominates in children. The response of the gluten-free diet was excellent in all of them, both from the digestive and the neurologic point of view in the average follow-up period of three years long.

The predominant neurologic clinical presentation on these celiac patients was myelitis in 4, and mixed in the rest. No ataxic forms were found. No specific findings were seen on their MRI's compared with non-celiac patients.

Only 3 celiac patients were treated with IFN beta 1-b, and we didn't find any more autoimmune disturbances in them. The remaining 5 CD patients, were only on a GFD.

We performed a family screening study in all the 72 MS patients, not only in the 8 celiacs, and we found a very high prevalence of CD among them, with a total of 23 first degree relatives positive among 126 screened (32%)

CD is also associated with several neurological conditions at a significantly higher frequency than in the general population, as can be observed in patients with migraine [32,33]. Cerebellar ataxia associated with gluten is the syndrome most commonly associated with CD, especially in adult patients. Many immunopathogenic mechanisms and different antibodies associated with gluten have been described which are capable of crossing the blood-brain barrier and deposit at the level of the Purkinje cells, where they produce a marked inflammatory response followed by neuronal degeneration and cerebellar atrophy [34,35]. An isoenzyme of TGt has been recently described, specifically the subtype 6, which is present in the cerebellum of patients with CD-associated ataxia, and its positivity would be useful in explaining the pathogenesis of this process [36].

## Conclusions

In this prospective series of 72 RRMS patients, we have found at least 8 (11.1%) with a related CD, confirmed by the presence of serological antibodies (TGt-2) in 7 and villous atrophy in all of them. This represents a prevalence of gluten intolerance 5.5 to 11 times higher in this group of patients than that found of the general population.

A gluten-free diet should be considered for any RRMS patient, who exhibits these serological and histological findings, characteristic of the presence of an associated celiac disease.

## Competing interests

The authors declare there are no competing interests.

## Authors' contributions

Conception and design: LR and CHL. The analysis and interpretation of the data: LR and SG. Drafting of the article: DF, NA, ALV, SG. Critical revision of the paper, for important intellectual content: LR, and CHL. All authors read and approved the final manuscript

## Pre-publication history

The pre-publication history for this paper can be accessed here:

http://www.biomedcentral.com/1471-2377/11/31/prepub
